# Meta-Analysis of Quantitative Trait Loci Associated with Seedling-Stage Salt Tolerance in Rice (*Oryza sativa* L.)

**DOI:** 10.3390/plants8020033

**Published:** 2019-01-29

**Authors:** Md. Shofiqul Islam, John Ontoy, Prasanta K. Subudhi

**Affiliations:** School of Plant, Environment, and Soil Sciences, Louisiana State University Agricultural Center, Baton Rouge, LA 70803, USA; islammdshofiqul@gmail.com (M.S.I.); jontoy@agcenter.lsu.edu (J.O.)

**Keywords:** consensus map, meta-QTL, *Oryza sativa*, salinity, shoot sodium content, sodium–potassium ratio

## Abstract

Soil and water salinity is one of the major abiotic stresses that reduce growth and productivity in major food crops including rice. The lack of congruence of salt tolerance quantitative trait loci (QTLs) in multiple genetic backgrounds and multiple environments is a major hindrance for undertaking marker-assisted selection (MAS). A genome-wide meta-analysis of QTLs controlling seedling-stage salt tolerance was conducted in rice using QTL information from 12 studies. Using a consensus map, 11 meta-QTLs for three traits with smaller confidence intervals were localized on chromosomes 1 and 2. The phenotypic variance of 3 meta-QTLs was ≥20%. Based on phenotyping of 56 diverse genotypes and breeding lines, six salt-tolerant genotypes (Bharathy, I Kung Ban 4-2 Mutant, Langmanbi, Fatehpur 3, CT-329, and IARI 5823) were identified. The perusal of the meta-QTL regions revealed several candidate genes associated with salt-tolerance attributes. The lack of association between meta-QTL linked markers and the level of salt tolerance could be due to the low resolution of meta-QTL regions and the genetic complexity of salt tolerance. The meta-QTLs identified in this study will be useful not only for MAS and pyramiding, but will also accelerate the fine mapping and cloning of candidate genes associated with salt-tolerance mechanisms in rice.

## 1. Introduction

Rice is a staple food for a majority of the world population. Among agricultural crops, it ranks third in worldwide production [1]. However, rice crop is exposed to many biotic and abiotic stresses. Soil and water salinity is one of the major abiotic stresses that significantly reduce rice production, not only in coastal areas but also in areas where rice production depends on artificial irrigation systems [2]. Rice plants are very sensitive to salt stress during seedling and reproductive stages and eventually die when the electrical conductivity (EC) reaches 10 dSm^−1^ [3]. Thus, the development of salt-tolerant varieties is a major breeding objective to increase the sustainability of rice farming.

To accelerate the development of salt-tolerant varieties, efforts have been made to identify large effect quantitative trait loci (QTLs) for plant survival, shoot and root lengths, Na^+^ and K^+^ uptake, and Na^+^/K^+^ ratio in both root and shoot under salt stress. As a result, hundreds of QTLs controlling salinity tolerance traits have been identified in different mapping populations [2,4,5,6,7,8,9,10,11,12,13]. The confidence intervals (CI) of these QTLs are variable, and numerous genes have been found within each QTL. However, our current knowledge of the gene networks that control the overall performance of rice plants under salt stress is still limited. The most promising QTL identified for salt tolerance during the seedling stage in rice is designated as *Saltol* QTL, which was mapped to the 10.7–12.2 Mb region on chromosome 1 explaining over 40% phenotypic variance for shoot Na^+^/K^+^ ratio [14,15]. Among the salt tolerance QTLs, only *SKC1* locus encoding a HKT-type Na^+^ transporter in the *qSKC1 QTL* was successfully isolated by map-based cloning on chromosome 1 [16]. The successful application of QTL information in breeding programs depends on the consistency of QTLs in different genetic backgrounds and environments, but the majority of them have been mapped in biparental populations that were evaluated in a single environment. Such QTLs may be of limited use for application in breeding programs due to their inconsistency and variability in multiple genetic backgrounds and multiple environments. Moreover, the efficacy of these QTLs may be hindered due to undesirable epistatic interactions in different genetic backgrounds [17,18]. Therefore, the information from previously identified QTL studies should be leveraged to enhance our understanding of the genetic mechanisms associated with salt tolerance [19]. 

Meta-analysis of QTLs is a computational technique to identify consensus QTLs and refine QTL positions on the consensus map from multiple mapping studies [20,21]. The QTLs identified by meta-analysis from a stack of QTLs at a confidence interval of 95% are called meta-QTLs (MQTLs), which further require validation using a set of germplasms or breeding lines. The MQTLs, with small CI, consistency, and large effect on a trait are useful for marker-assisted selection. To date, there have been few reports available on the meta-analysis of QTLs for yield-related traits [22,23] and grain yield under drought condition in rice [18]. Swamy et al. [18] utilized QTL mapping information from 15 mapping populations and delineated meta-QTLs for grain yield under drought stress to small chromosome segments. Some of these drought tolerance QTLs also corresponded to homologous regions harboring QTLs for grain yield under drought in other cereals such as maize, wheat, and barley. In another study [23], results from 82 studies in rice were integrated to delimit meta-QTLs for panicle-related traits to small physical and genetic intervals for use in marker-assisted selection and identify candidate genes for future studies. 

We report here results from the meta-analysis of QTLs for four traits associated with seedling-stage salt tolerance: the salt injury score (SIS), shoot sodium concentration (SNC), shoot potassium concentration (SKC), and shoot sodium–potassium ratio (SNK). The objectives of this study were as follows: (1) to develop a consensus map using simple sequence repeat (SSR) and single nucleotide polymorphism (SNP) markers; (2) to identify meta-QTLs for four traits related to seedling-stage salt tolerance and refine their genomic positions in rice; (3) to identify molecular markers in the meta-QTL regions for use in marker-assisted selection; and (4) to identify potential candidate genes present in the MQTL regions. 

## 2. Results

### 2.1. QTL Analysis of Traits Associated with Seedling-Stage Salt Tolerance 

**Figure 1:**
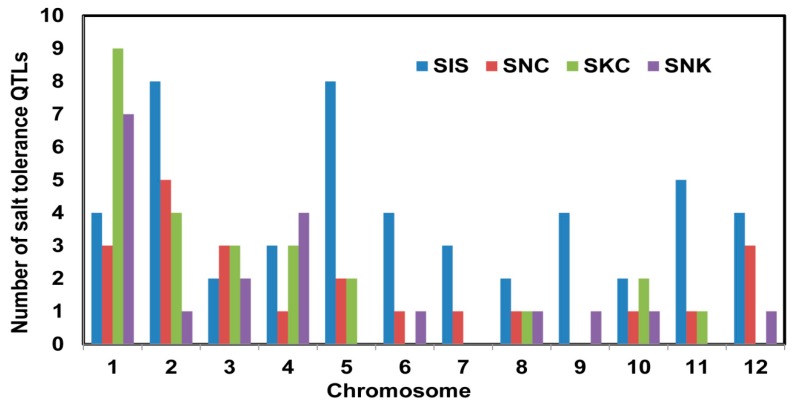
Number and distribution of initial quantitative trait loci (QTLs) for four salt-tolerance traits on 12 chromosomes in rice based on 12 QTL mapping studies. SIS, salt injury score; SNC, shot sodium concentration; SKC, shoot potassium concentration; SNK, shoot sodium–potassium ratio.

A total of 12 QTL mapping studies for seedling-stage salt tolerance were used in this study (Table 1) in which the mapping population size ranged from 62 to 285 lines. The number of markers in these studies ranged from 100 SSRs to 9303 SNPs. A total of 115 QTLs associated with salt tolerance attributes were reported, with the highest number of QTLs for SIS followed by SNC, SKC, and SNK. These QTLs were distributed on all chromosomes with a range of 3–27 QTLs per population (Figure 1). Chromosomes 7 had the lowest number of QTLs and chromosome 1 had the highest number of QTLs (Table S5). The distribution of QTLs showed that QTLs for all four traits associated with salt tolerance were located on chromosomes 1, 2, 3, 4, 8, and 10, whereas chromosomes 5, 6, 11, and 12 had QTLs for three traits, and chromosomes 7 and 9 had QTLs for two traits. The phenotypic variance explained by the initial QTLs varied from 0.13% to 41% and the confidence interval (CI) of markers varied from 0.31 to 72 cM. After integrating all maps, there were 12,327 SSR and SNP markers on the consensus map with a total map length of 2936 cM and a mean distance of 0.24 cM between the markers (Table S5, Figure S1).

### 2.2. Meta-Analysis of the QTLs

**Figure 2:**
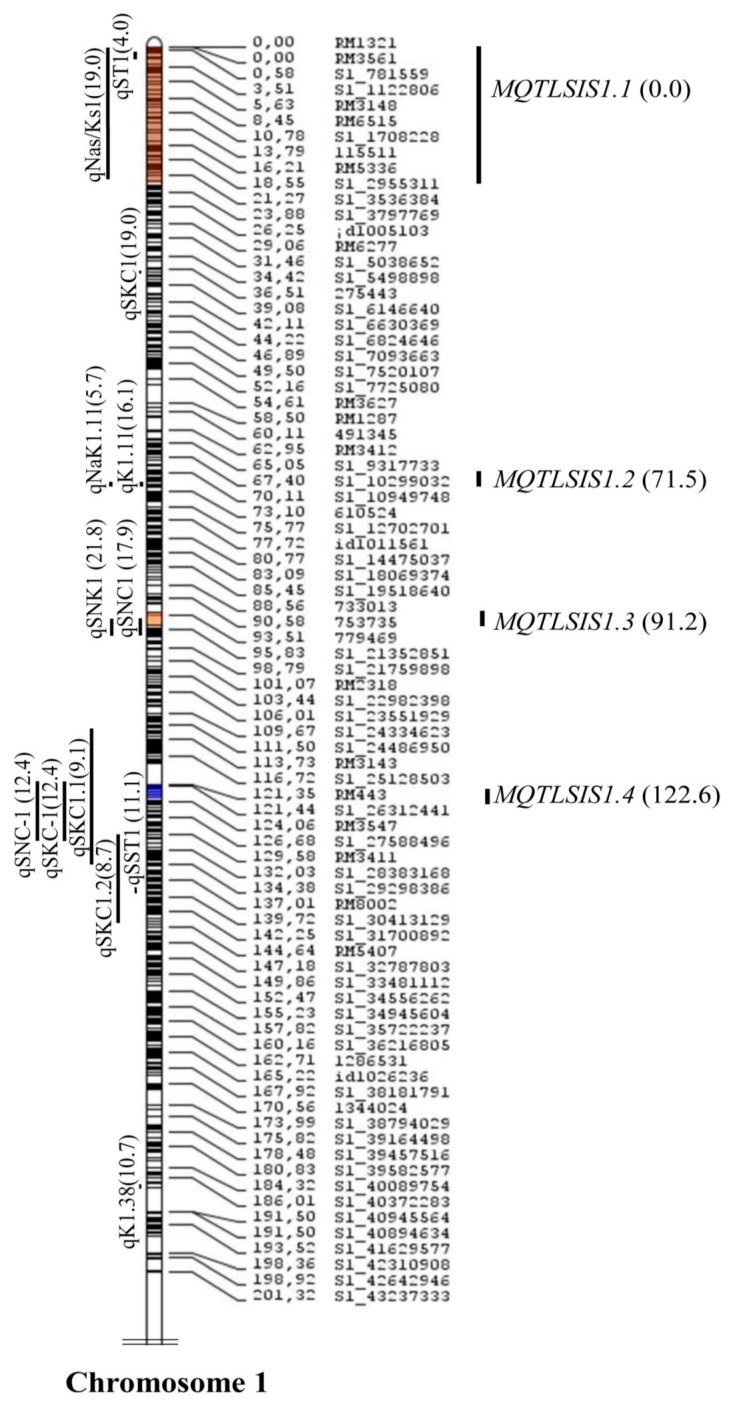
Meta-QTLs for salt injury score (SIS) in rice on chromosome 1. Vertical lines on the left of the chromosomes show the confidence interval of each QTL and values in parentheses indicate the percentage of phenotypic variation explained by these QTLs. Four meta-QTLs for SIS were shown on the right of the chromosomes, with the confidence interval indicated by vertical bars and the peak position of the meta-QTLs in cM on the consensus map (in parenthesis).

**Figure 3:**
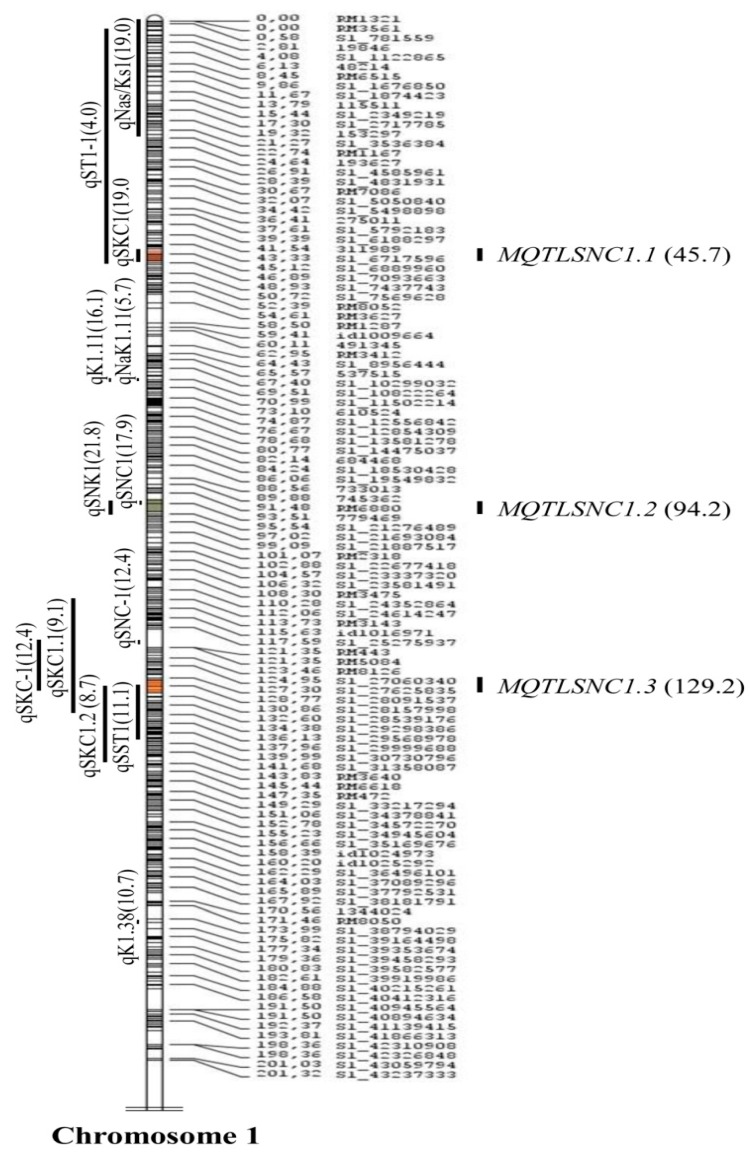
Meta-QTLs for shoot sodium concentration (SNC) in rice on chromosome 1. Vertical lines on the left of the chromosomes show the confidence interval of each QTL and values in parentheses indicate the percentage of phenotypic variation explained by these QTLs. Four meta-QTLs for SIS were shown on the right of the chromosomes, with the confidence interval indicated by vertical bars and the peak position of the meta-QTLs in cM on the consensus map (in parenthesis).

**Figure 4:**
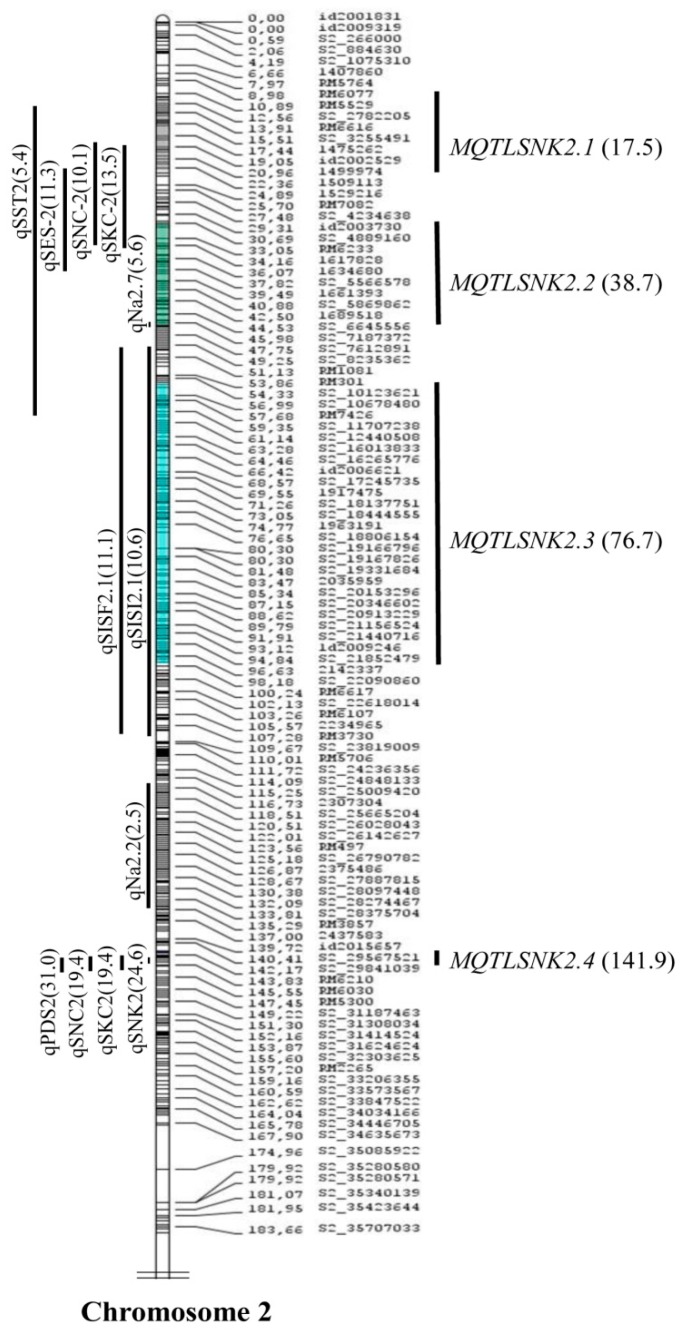
Meta-QTLs for shoot sodium potassium ratio (SNK) in rice on chromosome 2. Vertical lines on the left of the chromosomes show the confidence interval of each QTL and values in parentheses indicate the percentage of phenotypic variation explained by these QTLs. Four meta-QTLs for SIS were shown on the right of the chromosomes, with the confidence interval indicated by vertical bars and the peak position of the meta-QTLs in cM on the consensus map (in parenthesis).

A total of 11 meta-QTLs were identified for SIS, SNC, and SNK (Table 2). The meta-QTLs were identified with a CI of 95% on chromosomes 1 and 2 based on the lowest Akaike information criterion (AIC) values (Figure 2, Figure 3 and Figure 4). A total of seven meta-QTLs for SIS and SNC and four for SNK were located on chromosomes 1 and 2, respectively. Among them, two meta-QTLs, *MQTLSIS1.3* and *MQTLSNC1.2*, co-localized at the 94.2 cM position on chromosome 1. The phenotypic variance of the meta-QTL varied from 9% to 24%. The mean phenotypic variance was ≥10% for 9 meta-QTLs (Table 2).

**Figure 5:**
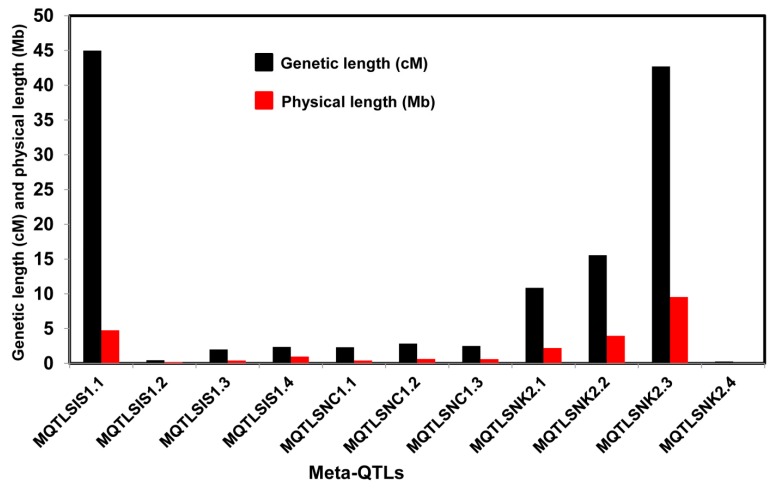
Genetic and physical length of the meta-QTLs. Black bars demonstrate the genetic length (cM) and red bars indicate the physical length (Mb) of the meta-QTLs.

The CI of all meta-QTLs was smaller than their respective initial QTLs. It varied from 0.28 cM on chromosome 2 to 45 cM on chromosome 1 (Table 2). In the cases of *MQTLSIS1.1* and *MQTLSNK2.3*, the CIs were 45 cM and 43 cM, respectively. The physical length of the meta-QTLs ranged from 0.04 to 9.54 Mb (Figure 5). Among the meta-QTLs, seven meta-QTLs had less than 1.0 Mb intervals with a maximum genetic map distance of 3 cM and phenotypic variances of more than 10% (Figure 2, Figure 3 and Figure 4, Table 2). There were 4 QTLs for SIS and 3 QTLs for SNC on chromosome 1 and three for SNK on chromosomes 2. Four meta-QTLs were located on less than a 500 kb region and one of these, *MQTLSNK2.4*, had ~37 kb CI with a phenotypic variance of more than 20% (Table 2).

### 2.3. Identification of Genes in the Meta-QTL Regions

All 11 meta-QTL regions contain a total of 2938 genes, with the lowest (31) and highest (1495) number of genes in *MQTLSIS1.2* and *MQTLSNK2.3* intervals, respectively (Table S6). Approximately, over 70% of the genes were hypothetical proteins (173), expressed proteins (815), transposons (692) and retrotransposons (469). The remaining 30% were putative genes/gene families localized in the meta-QTL intervals. Only 265 genes were differentially expressed in the salt-tolerant genotypes (Table S6), when compared with the available expression data of salt stress studies [26,27]. These genes/gene families include cytochrome P450, expressed protein, hypothetical protein, zinc finger protein, protein kinase, protein phosphatase 2C, receptor-like protein kinase precursor, transcription factor like protein, phytosulfokine receptor, transporter like protein, transferase like protein, F-box, hydrolase like protein, leucine zipper protein, and DNA domain containing protein.

### 2.4. Gene Ontology (GO) Analyses

The result of the GO analysis (Table S7) showed that 1272 of the 2938 genes were functionally annotated using the AgriGO v.2.0 database. The annotated gene number varied from 0 (*MQTLSIS1.2*) to 614 (*MQTLSNK2.3*). There were no functionally annotated genes in *MQTLSIS1.2*, possibly due to the lower number of genes present in this region. Only 5 of the 11 meta-QTLs had a total of 47 significant GO terms. The percentage of significant GO terms varied from 0.5 to 56.3. The *MQTLSIS1.4* had only two significant GO terms—GO: 0008168 (methyltransferase activity) and GO: 00016741 (transferase activity) under molecular function (Table S8). The *MQTLSNK2.1* had 16 significant GO terms such as metabolic and biosynthetic processes under the biological process, and 12 significant GO terms including protein binding, transmembrane transporter activity, and hydrolase activity. There were three significant GO terms associated with cell wall organization under the biological process and two significant GO terms for protein binding and nutrient reservoir activity in *MQTLSNK2.2*. Similarly, the *MQTLSNK2.3* had three significant GO terms for transferase activity and protein serine/threonine phosphatase activity, whereas it had nine significant GO terms for cell and organelle under cellular components for *MQTLSNK2.4*. There were no significant GO terms for the meta-QTLs for SNC. 

### 2.5. Phenotyping and Genotyping of Rice Genotypes Using Meta-QTL-Linked Markers

Based on phenotyping data, six genotypes—Bharathy, Langmanbi, I Kung Ban 4-2 Mutant, Fatehpur 3, CT-329, and IARI 5823—were identified as salt tolerant. These genotypes had low SIS scores and low Na^+^/K^+^ ratios (Table S3). The salt-tolerant donors, Pokkali, Nona Bokra, and FL478 showed a salt tolerance response as expected with low SIS scores and low Na^+^/K^+^ ratios, whereas IR29, Cheniere, and Jupiter were highly susceptible to salt stress. Most of the selected breeding lines, which were developed using the donor ‘Nona Bokra’, showed improved salt tolerance compared to their corresponding recurrent parents Jupiter and Cheniere [8,25].

Twelve polymorphic SSR markers were selected in the 9 meta-QTL regions for the meta-QTL validation (Figure S2). All primers were located within 5 Mb regions of the reported salt-tolerant genes such as WRKY10 (*LOC_Os01g09100*), receptor-like protein kinase 5 precursor, putatively expressed (*LOC_Os01g53920*), chloride transporter (*LOC_Os01g50860*), and zinc finger protein gene (*LOC_Os01g15630*). RM3411 was in close proximity of serine/threonine protein kinase (*LOC_Os01g54480*). On chromosome 2, the genes in the neighborhood of SNK meta-QTLs were protein kinase, putatively expressed (*LOC_Os02g06930*), MYB family transcription factor, putatively expressed (*LOC_Os02g07170*), Na^+^ transporter (*LOC_Os02g07830*), chloride channel protein (*LOC_Os02g35190*), and MYB family transcription factor (*LOC_Os02g53670*). There were also several transcription factor genes located close to the selected markers. 

Genotyping results also revealed that there were multiple alleles for some of these SSR loci, and 16 genotypes were heterozygous for at least one locus (Table S9). Although most salt-tolerant breeding lines derived from mapping populations involving Nona Bokra inherited the salt-tolerant alleles, none of the molecular markers could distinguish rice genotypes based on the level of salt tolerance. The alleles of SSR loci present in salt-tolerant accessions were also present in salt susceptible accessions. 

## 3. Discussion

The complex quantitative traits are influenced by both genetic and environmental factors. Many agronomic traits in plants, including salt tolerance, are complex traits controlled by QTLs [16]. The analysis of QTLs is often conducted to understand the molecular genetic basis of variation observed in those polygenic traits [23]. Although numerous QTLs have been identified for salt-stress tolerance in rice using different salt-tolerant donors [2,7,8,10,28,29,30], the success in the introgression of these QTLs in rice-breeding programs is limited due to the inconsistency of QTLs in different genetic backgrounds and environments [31]. However, the cloning of the *SKC1* locus corresponding to the QTL *qSKC1* [16] indicated that QTLs can be mapped accurately when the genetic distances between the flanking markers are 2 cM or less [31]. Therefore, efforts should be made to identify the most accurate and precise major-effect QTLs associated with salinity tolerance across different genetic backgrounds and environments to make progress through marker-assisted breeding. The meta-QTL approach provides an opportunity to utilize QTL information from multiple mapping populations with different genetic backgrounds for the precise location of QTLs. 

### 3.1. Meta-QTL Analysis of Salt Tolerance in Rice

Independent genetic mapping studies have generated many QTLs for salt tolerance-related traits. The map positions of these QTLs are variable due to use of diverse salt-tolerant donors in mapping populations. Therefore, combining all QTL information in a particular region of the genome is done through QTL meta-analysis to identify precise QTL locations [32]. Several studies identified precise meta-QTLs for various traits in rice, wheat, barley, perennial ryegrass [22,23,33,34,35,36,37] to mine candidate genes for yield, panicle-related traits, root genetic architecture, and abiotic-stress tolerance such as drought, salinity, and waterlogging. In this study, meta-QTL analysis was conducted for a few salt tolerance-related traits—SIS, SNC, SKC, and SNK—in rice. Although the initial 115 QTLs related to various salt-tolerance traits were spread on all 12 chromosomes (Figure 1), emphasis was given to three important traits—SIS, SNC, and SNK—and 11 meta-QTLs on chromosomes 1 and 2 were identified (Figure 2, Figure 3 and Figure 4). A study by Zheng, et al. [13] identified QTLs for the SNC and SKC (*qSNC-1* and *qSKC-1* on chromosome 1; *qSNC-2* and *qSKC-2* on chromosome 2) using the same SSR flanking markers, suggesting the same chromosomal regions control both SNC and SKC. Four meta-QTLs were identified for SIS, which has been widely used to evaluate the overall performance of rice genotypes for seedling-stage salt tolerance. SIS scoring is dependent on Na^+^ and K^+^ uptake by plants during salt stress. In earlier studies, QTLs for the correlated traits clustered on the same chromosomal regions [7,8,13]. This trend was also observed in this meta-QTL study. For example, two meta-QTLs (*MQTLSIS1.3* and *MQTLSNC1.2*) co-localized at the 94.2 cM position on chromosome 1 (Table 2). The co-localization of meta-QTLs for different traits may be due to tight linkage of genes or pleiotropy [38]. 

A previous study identified *Saltol* QTL using amplified fragment length polymorphism (AFLP) markers at the position of 10.7–12.2 Mb on chromosome 1 in the mapping population developed from the cross IR29 × Pokkali [15]. A follow-up study confirmed that the *Saltol* region for shoot Na^+^/K^+^ ratio explained 43% of phenotypic variation in 54 recombinant inbred lines (RILs) [14]. Later, SSR markers were used to characterize the *Saltol* QTL at the seedling stage using the same IR29/Pokkali mapping population, and a significant QTL for Na^+^/K^+^ ratio was found in the *Saltol* region, explaining 27% of the phenotyping variation [10]. Two meta-QTLs, *MQTLSNC1.1* (8.52–8.94 Mb) and *MQTLSIS1.2* (14.07–14.27 Mb) were located in close proximity of the *Saltol* region, which confirmed that meta-analysis is more informative than individual studies and can give greater insight into the genetic architecture of complex traits [39]. 

### 3.2. Gene Content in the Meta-QTL Regions

The meta-analysis of QTLs followed by the mining of genes in the meta-QTL intervals is a suitable alternative to fine mapping for the identification of candidate genes. The analysis of the gene content of *MQTLSIS1.1* revealed the presence of two transcription factor (TF) genes, *WRKY107* (*LOC_Os01g09080*) and *WRKY10* (*LOC_Os01g09100*), and a histone-like transcription factor and archaeal histone gene (*LOC_Os01g08790*) (Table S6). These genes may play a role in salt tolerance. The second SIS meta-QTL, *MQTLSIS1.2,* contained the candidate gene dirigent (*LOC_Os01g24960* and *LOC_Os01g25030*), which is involved in lignification and plays a pivotal role against biotic and abiotic stresses in plants [40]. The SIS meta-QTL *MQTLSIS1.3* and SNC meta-QTL *MQTLSNC1.2* were identified at the same location (22.25-22.88 Mb) of chromosome 1 (Table 2), where a shoot Na^+^ concentration QTL (*qSNC1*) explaining 18% phenotypic variation was mapped earlier [7]. This region was found to contain several putative genes involved in apoptosis, signal transducer activity, transcription factor activity, and translation factor activity. Under salt stress, genes encoding programmed cell death protein (*LOC_Os01g39650*), protein kinase domain containing protein (*LOC_Os01g39970*), protein phosphatase 2C (*LOC_Os01g40094*), eukaryotic translation initiation factor 5B (*LOC_Os01g40150*), translation initiation factor (*LOC_Os01g40170*), WRKY77 (*LOC_Os01g40260*), integral membrane protein (*LOC_Os01g40280*) and amino acid transporter family protein (*LOC_Os01g40360*), were activated suggesting their significant role in regulation of shoot Na^+^ uptake and overall performance. Earlier studies [7,41] have shown that *MQTLSIS1.3* and *MQTLSNC1.2* overlapped with the OsPP2C06 gene (*LOC_Os01g40094*), which is up-regulated by the over-expression of a family member of the abiotic stress-inducible NAC transcription factor [42]. Another salinity responsive transcription factor gene, *OsWRKY77* 1 [7,43], was identified within the *MQTLSNC1.2* interval (Table S6). Similarly, in the *MQTLSIS1.4* region, several candidate genes encoding serine/threonine protein phosphatase family protein (*LOC_Os01g49690*), zinc finger (C3HC4-type) domain containing protein (*LOC_Os01g49770* and *LOC_Os01g50750*), protein kinase (*LOC_Os01g49920*), ABC transporter-like (*LOC_Os01g50080, LOC_Os01g50100* and *LOC_Os01g50160*), MYB family transcription factor (*LOC_Os01g50110* and *LOC_Os01g50720*), transporter facilitator family (*LOC_Os01g50820*), and helix-loop-helix DNA-binding domain containing protein (*LOC_Os01g50940*) were identified. The contribution of C3HC4- type zinc finger proteins toward salt stress tolerance in rice has been reported [44]. The ABC transporter genes are known to play an important role in salt tolerance mechanism in rice by acting as transmembrane transporters [45]. Another gene, *LOC_Os01g50860*, encoding a chloride transporter, found in the *MQTLSIS1.4* region, might be a promising candidate for salt stress tolerance due to its involvement in ion homeostasis and transport processes. The *LOC_Os03g48940*, encoding a chloride channel protein CLC-d, was reported as a salinity-responsive candidate gene [41].

Several genes were found within the *MQTLSNC1.1* region in this study. Among them, *LOC_Os01g15470* and *LOC_Os01g15630* encoding protein kinase and C3HC4-type zinc finger domain containing protein, respectively, were the most likely candidates for salt tolerance in rice. Among the annotated genes within the *MQTLSNC1.3* region, a serine/threonine protein kinase gene (*LOC_Os01g54480*) was reported to improve salt tolerance through modulation of several abiotic stress related genes in rice [46]. A receptor like kinase (RLK) gene (*OsSIK1*) containing leucine-rich repeats [47] and a cysteine-rich repeat RLK sub-family gene (*ARCK1*) were induced by drought and salt stress in rice [48].

The genes found within the meta-QTL for shoot Na^+^/K^+^ were involved in ion homeostasis, transport processes, signal transducer activity, transcription factor, and translation factor activity. The notable genes involved in sodium homeostasis were *OsHKT1;3* Na^+^ transporter (*LOC_Os02g07830*) in *MQTLSNK2.1* region and potassium transport related genes (*LOC_Os02g31910* and *LOC_Os02g31940*) in *MQTLSNK2.3. OsHKT1;3* is a highly selective Na^+^ transporter in leaves and roots [49], which may contribute to regulate Na^+^ in rice leaf blades during salt stress. The potassium channel KAT1 (*LOC_Os02g14840*) and a serine/threonine protein kinase gene (*LOC_Os02g34430*) located within *MQTLSNK2.2* and *MQTLSNK2.3* regions, respectively, might be responsible for saline tolerance rice through regulation of multiple genes involved in abiotic stress tolerance. A gene encoding chloride channel protein (*LOC_Os02g35190*) and a salt-tolerant protein gene *LOC_Os02g35880* were present within the *MQTLSNK2.3* interval. Similarly, *LOC_Os02g15580* and *LOC_Os02g35830,* encoding the cyclic nucleotide-gated ion channel and extracellular ligand-gated ion channel, were located within the *MQTLSNK2.2* and *MQTLSNK2.3*, respectively. Further, genes such as cation efflux family protein (*LOC_Os02g53490*), MYB family transcription factor (*LOC_Os02g53670*) and growth regulating factor protein (*LOC_Os02g53690*) located within the *MQTLSNK2.4* region might be potential candidates for salt tolerance mechanisms in rice. A detailed investigation of these above genes in the meta-QTL regions would provide valuable information to enhance our understanding of the salt tolerance mechanisms in rice.

### 3.3. Selection of Salt-Tolerant Germplasm Using Meta-QTL Linked Markers

The level of salt tolerance in six salt-tolerant genotypes (Bharathy, I Kung Ban 4-2 Mutant, Langmanbi, Fatehpur 3, CT-329, and IARI 5823) identified in this study was comparable to the two well-known salt tolerant donors, Pokkali and Nona Bokra (Table S3). In general, meta-QTLs with a small CI can accelerate fine mapping, candidate gene identification, functional analyses, and MAS. In this study, the confidence intervals of QTLs were reduced drastically in some meta-QTL regions such as *MQTLSIS1.2, MQTLSIS1.4, MQTLSNC1.1, MQTLSNC1.3,* and *MQTLSNK2.4*. The physical length of the meta-QTL intervals was variable. This variability in QTL intervals could be attributed to the quality of marker data and QTL mapping information obtained from various studies. Our failure to use the meta-QTL linked markers to distinguish salt-tolerant genotypes from salt-susceptible ones could be due to the following reasons. Associating molecular markers linked to meta-QTL is challenging due to the genetic complexity of multiple salt tolerance mechanisms controlled by many genes. First, it is well known that none of the salt-tolerant donors has all the desirable alleles for all salt tolerance mechanisms. On the other hand, a salt-susceptible genotype may possess desirable alleles, but their cumulative effect may not be adequate to exhibit a salt tolerance response. The second reason could be the poor resolution of the meta-QTLs identified in this study, and the markers used for genotyping were therefore loosely linked to salt-tolerance genes. For example, some selected salt-tolerant breeding lines developed using the ‘Nona Bokra’ used in this study have different alleles of some microsatellite loci, which might have arisen due to recombination. The highly susceptible ‘IR29’ also had salt-tolerant Pokkali and Nona Bokra alleles in some SSR loci. The inclusion of parents of the mapping populations and use of a large set of genotypes might have been helpful. Despite these bottlenecks, the findings from this study suggest that the fine mapping of these meta-QTLs using markers developed from the candidate genes located in the meta-QTL intervals can be useful for marker-assisted breeding to improve salt tolerance in rice.

### 3.4. Future Perspectives

The overall goal of genomic and genetic studies in crop plants is to elucidate the molecular basis of agronomically important traits to improve crop quality and productivity using germplasm resources such as elite breeding lines, land races, mutants and wild relatives. Recent advances in high-throughput whole-genome sequencing technologies have accelerated genomic research by drastically reducing the cost of genome-wide variant discovery in many crop species. The integration of the whole genome re-sequencing data with the QTL information will be helpful for the discovery of genes underlying complex agronomic traits. The overlapping of some meta-QTLs such as *MQTLSIS1.3* and *MQTLSNC1.2* with a major QTL for shoot Na^+^ concentration QTL [7] demonstrates the reliability of the meta-analysis of QTLs. The meta-QTL regions for the salt-tolerance traits identified in this study can be compared between salt-susceptible and salt-tolerant genotypes to identify the genes and their variants to improve our current knowledge about the complex salt tolerance mechanisms in rice.

## 4. Materials and Methods

### 4.1. Data Collection and Input File Preparation

Twelve published QTL mapping studies for seedling-stage salt tolerance were selected (Table 1), in which detailed information of the genetic maps, including parents, types of mapping population, population size, number and type of marker and genetic distances in the linkage groups were available. Four traits associated with salt tolerance—SIS, SNC, SKC, and SNK—were analyzed in this study. Those QTLs with available map positions, logarithm of odds (LOD) scores, and R^2^ values were integrated for analysis. The QTL studies with any missing parameters were discarded. Two types of input data text files were prepared from each study according to the instruction manual of BioMercator v3/v4 [20]. One was genetic map file and the other was for QTL information. The headers of each input file were given in the BioMercator’s user guide.

### 4.2. Construction of Consensus Map and Projection of QTLs

A consensus genetic map was constructed to perform QTL meta-analysis using Biomercator v4.2 [20]. The SSR-based rice genetic linkage map from the International Rice Microsatellite Initiative 2003 [50] and SNP-based high-density linkage maps [2,7] were integrated as high-density reference maps (Table S1), on which the markers from all studies were projected to develop an integrated consensus map (Table S2).

Before projecting the QTLs on the consensus map, the 95% CI of the initial QTL on their original genetic map was calculated [51]. The 95% CI was computed as $\mathrm{CI}=\frac{530}{N\lambda }$, where λ is the proportion of phenotypic variance explained by the QTL and N is the population size. The CI was calculated to ensure consistency in different studies. The QTL positions on the consensus chromosome map was projected using a scaling rule between the marker interval of the original QTL and the corresponding interval on the consensus chromosome [20]. The new CI of a QTL on the consensus linkage group was computed using Gaussian distribution [20].

### 4.3. Meta-Analysis of QTLs

After projecting the QTLs on the consensus linkage group, meta-analysis was performed on the QTL clusters using Veyrieras’s algorithm for each trait independently on each chromosome using the default parameter settings of BioMercator v4.2 [20]. The lowest AIC value was used to select the best QTL model for each chromosome, which was considered significant to identify a number of meta-QTLs.

### 4.4. Identification of Genes within the Meta-QTL Regions

The rice genome with structural and functional annotation from MSU rice 7.0 database and anchor markers with their physical position that connect between the genetic map and the sequences [52] were loaded in BioMercator according to the software’s instruction to display the genomic regions of the meta-QTLs. The precise genetic length of each meta-QTL was used to mine the genes present in these 11 meta-QTL regions from the annotated gene information stored in the BioMercator software. Then, the gene content within each meta-QTL interval was exported from BioMercator as GFF3 file, which contains both QTLs and MQTLs information.

### 4.5. Gene Ontology (GO) Enrichment Analysis

The functional classification of the genes within the meta-QTL regions was performed by singular enrichment analysis (SEA) tool using the web-based AgriGO v2.0 (http://systemsbiology.cau.edu.cn/agriGOv2/) [53] with the following parameter settings: (i) Fisher’s exact test with the Benjamini–Yekutieli (false discovery rate under dependency) multiple test adjustment method, and (ii) significance level α = 0.05. Singular enrichment analysis was used to identify the GO terms, namely biological processes, molecular functions, and cellular components that are significantly enriched by identified expressed genes for each of the traits. MSU7.0 gene ID (e.g., *LOC_Os06g29340*) was used as reference during SEA analysis.

### 4.6. Salt Tolerance Screening and Marker Profiling in Rice Genotypes

A total of 56 diverse genotypes consisting of 42 accessions from National Genetic Resources Program (NGRP), 3 known salt-tolerant donor lines, 3 known salt-sensitive varieties, and 8 selected advanced salt-tolerant breeding lines (Table S3) were screened in a greenhouse for seedling-stage salinity tolerance following the protocol developed at the International Rice Research Institute with minor modifications [54]. A randomized complete block design with 2 replications was followed. All lines were germinated in Petri dishes in the laboratory and were transferred to nutrient solution containing 200 mg/L ferrous sulfate and 1 g/L of Jack’s professional fertilizer 20-20-20 (J.R. Peters, Inc.). After 14 days, they were subjected to a salt stress level of 6 dSm^−1^ by adding NaCl to the nutrient solution for 2 days, followed by exposure to 12 dSm^−1^ salt stress. The pH of the nutrient solution was adjusted to 5.0. The plants were grown for ~10 days at 12 dsm^−1^ salt stress before recording the salt injury score (SIS) through visual inspection (Figure S3). When the susceptibility check IR29 showed the characteristic salt injury symptoms, three uniform plants from each genotype were selected for SIS scoring on a scale of 1–9. A score of 1 indicates that the line is highly tolerant, and a score of 9 indicates high susceptibility. After screening, tissues from each genotype were oven-dried at 65 °C for 10 days followed by homogenization. One hundred milligrams of tissue was digested with nitric acid—hydrogen peroxide (5:3 mL) in a 152–155 °C heating block for 3 h [55]. A flame photometer (model PFP7, Bibby Scientific Ltd., Staffordshire, UK) was used to measure the total amount of Na^+^ and K^+^. The final concentrations of Na^+^ and K^+^ ions were computed using a standard curve.

For genotyping, the DNA was extracted from fresh leaf samples of all rice genotypes using a modified CTAB method [56]. DNA samples were quantified on a 1.5% agarose gel and the concentration was adjusted to approximately 50 ng μL^−1^. PCR amplification was done with a 15 μL reaction mixture having 50 ng DNA, 10X PCR buffer, 100 μM dNTPs, 250 μM primers, and 1U of Taq polymerase. The PCR profile was as follows: an initial denaturation of DNA at 95 °C for 7 min, followed by 35 amplification cycles of denaturation at 95 °C for 45 sec, annealing temperatures varied from 55 °C to 58 °C for 45 s based on the primer, extension at 72 °C for 1 min and final extension at 72 °C for 7 min. The PCR products were resolved on 8% non-denaturing polyacrylamide gels. The gels were scored based on donor alleles as reference bands for QTL validation. Since the SSR markers linked to *MQTLSIS1.3* and *MQTLSNC1.2* were not polymorphic (Figure S2), 12 polymorphic SSR markers linked to 9 meta-QTLs were selected. The sequences were obtained from the markers database in Gramene (http://archive.gramene.org/markers/) and were synthesized from the Integrated DNA Technologies, Coralville, IA, USA (Table S4).

## Figures and Tables

**Table 1:** The QTL mapping studies used for meta-QTL analysis for traits associated with seedling-stage salt tolerance.

Mapping Population			Markers Used		QTL Identified ^$^				References
Parents	Type	Size	Type	Number	SIS	SNC	SKC	SNK	
Teqing × Tarome-Molaei	BC_2_F_5_	62	SSR	114	-	-	2	1	[24]
IR29 × Hasawi	RIL	142	SNP	194	5	-	-	-	[4]
Bg352 × At354	RIL	100	SSR/InDel	158	2	-	-	2	[5]
Bengal × Pokkali	RIL	187	SNP	9303	7	3	2	2	[2]
Bg352 × At354	RIL	94	SNP	1135	8	7	5	7	[7]
Jupiter × Nona Bokra	IL	138	SSR	126	4	4	4	2	[8]
Cheniere × Nona Bokra	IL	112	SSR	116	5	1	3	3	[25]
Ce258 × IR75862	BC_1_F_10_	200	SSR	128	4	1	3	-	[9]
Zhongguangxiang1 × IR75862	BC_1_F_10_	200	SSR	133	2	1	2	-	[9]
IR29 × Pokkali (Experiment 1)	RIL	140	SSR	100	2	1	1	2	[10]
93-11 × *O. rufipogon*	IL	285	SSR	142	8	-	-	-	[12]
Dongnong425 × Changbai10	BC_2_F_2_	190	SSR	137	2	4	3	-	[13]
Total				11,786	49	22	25	19	

**^$^** SIS, salt injury score; SNC, shoot sodium concentration; SKC, shoot potassium concentration; SNK, shoot sodium–potassium ratio.

**Table 2:** Meta-QTLs for seedling-stage salt tolerance traits in rice.

Trait	Meta-QTLs	Ch	AIC Value	QTL Model	Marker Interval	Meta-QTL Peak Position (cM)	Physical Position (Mb)	No of Initial QTL	Mean Phenotypic Variance of the QTL (%) ^$^	Mean Initial CI (cM)	Meta-QTL CI (95%) (cM)	Physical Length of Meta-QTL (Mb)
SIS	*MQTLSIS1.1*	1	72.6	4	RM1321-RM1167	0	0.003–4.75	2	11.5	45.75	44.99	4.749
	*MQTLSIS1.2*				S1_11502301-S1_11584932	71.5	14.07–14.27	2	10.9	0.64	0.45	0.198
	*MQTLSIS1.3*				774607-RM5853	94.17	22.33–22.75	2	19.9	2.86	2.01	0.415
	*MQTLSIS1.4*				RM443-S1_26769954	122.57	28.34–29.32	3	11.3	13.96	2.37	0.983
SNC	*MQTLSNC1.1*	1	109.2	3	S1_6824646-S1_7093663	45.68	8.52–8.94	2	11.5	24.4	2.32	0.415
	*MQTLSNC1.2*				RM6880-S1_21352851	94.17	22.25–22.88	2	19.9	2.86	2.85	0.631
	*MQTLSNC1.3*				S1_27841959-S1_28157998	129.15	30.95–31.56	5	10.7	12.04	2.51	0.604
SNK	*MQTLSNK2.1*	2	92.1	4	S2_2747069-S2_3978527	17.45	2.44–4.66	3	9.7	26.04	10.87	2.207
	*MQTLSNK2.2*				S2_4889160-S2_7221617	38.74	5.90–9.88	4	10.1	23.42	15.57	3.973
	*MQTLSNK2.3*				RM3178-S2_22090860	76.68	14.99–24.53	3	9.0	55.32	42.7	9.541
	*MQTLSNK2.4*				RM3302-S2_29841039	141.96	32.89–32.93	4	23.7	2.22	0.28	0.037

Ch, chromosome; Mb, Mega base; AIC, Akaike information criterion; CI: confidence interval; ^$^ Mean phenotypic variance of the QTL was calculated among the initial QTLs that produced a meta-QTL.

## Supplementary Materials

The following are available online at http://www.mdpi.com/2223-7747/8/2/33/s1, Figure S1: The integrated consensus map developed by Biomercator. The consensus map contained 12,327 markers (most of them are hidden; the whole marker set were given in the Supplementary Table S2) covering a length of 2935.9 cM and 115 QTLs were projected on it. The names of the QTLs were given in the left side of each chromosome. Vertical and horizontal lines on the left of the chromosomes show the confidence interval of each QTL and variance, respectively. Markers and genetic distance (cM) were given on the right side of each chromosome; Figure S2: Physical location of markers (red color) linked to the meta-QTLs listed in Table S4 and nearby candidate genes within the identified meta-QTL regions. The physical positions were shown as MB in left side of the chromosome bar; Figure S3: Seedling screening for SIS, SNC, SKC and SNK of rice genotypes in hydroponic experiment. A. Control, B. Replication 1, and C. Replication 2; Table S1: High density reference map based on the mapping information of SSR [50] and SNP markers [2,7]; Table S2: Integrated consensus map; Table S3: List of rice germplasm and breeding lines used for meta-QTL validation; Table S4: Details of SSR markers used for meta-QTL validation; Table S5: Summary of the integrated consensus map; Table S6: List of genes identified in the six meta-QTL regions; Table S7: Gene ontology enrichment analyses of the meta-QTLs regions related to seedling stage salt tolerance traits; Table S8: List of significant GO terms obtained in the five meta-QTL regions for seedling stage salt tolerance; Table S9: A summary of genotyping results in rice germplasm and breeding lines using all linked SSR markers associated with meta-QTLs identified in this study.

## References

[1] FAOSTAT Crops/Regions/World list/Production Quantity/Rice (Paddy) in 2014. http://www.fao.org/faostat/en/#data/QC.

[2] De Leon T.B., Linscombe S., Subudhi P.K. (2016). Molecular dissection of seedling salinity tolerance in rice (*Oryza sativa* L.) using a high-density GBS-based SNP linkage map. Rice.

[3] Munns R., James R.A., Läuchli A. (2006). Approaches to increasing the salt tolerance of wheat and other cereals. J. Exp. Bot..

[4] Bizimana J.B., Luzi-Kihupi A., Murori R.W., Singh R.K. (2017). Identification of quantitative trait loci for salinity tolerance in rice (*Oryza sativa* L.) using IR29/Hasawi mapping population. J. Genet..

[5] Dahanayaka B.A., Gimhani D., Kottearachchi N., Samarasighe W.L.G. (2017). QTL mapping for salinity tolerance using an elite rice (*Oryza sativa*) breeding population. SABRAO J. Breed. Genet..

[6] De Leon T.B., Linscombe S., Subudhi P.K. (2017). Identification and validation of QTLs for seedling salinity tolerance in introgression lines of a salt tolerant rice landrace ‘Pokkali’. PLoS ONE.

[7] Gimhani D.R., Gregorio G.B., Kottearachchi N.S., Samarasinghe W.L.G. (2016). SNP-based discovery of salinity-tolerant QTLs in a bi-parental population of rice (*Oryza sativa*). Mol. Genet. Genom..

[8] Puram V.R.R., Ontoy J., Linscombe S., Subudhi P.K. (2017). Genetic dissection of seedling stage salinity tolerance in rice using introgression lines of a salt tolerant landrace Nona Bokra. J. Hered..

[9] Qiu X., Yuan Z., Liu H., Xiang X., Yang L., He W., Du B., Ye G., Xu J., Xing D. (2015). Identification of salt tolerance-improving quantitative trait loci alleles from a salt-susceptible rice breeding line by introgression breeding. Plant Breed..

[10] Thomson M.J., de Ocampo M., Egdane J., Rahman M.A., Sajise A.G., Adorada D.L., Tumimbang-Raiz E., Blumwald E., Seraj Z.I., Singh R.K. (2010). Characterizing the *Saltol* quantitative trait locus for salinity tolerance in rice. Rice.

[11] Thomson M.J., Tai T.H., McClung A.M., Lai X.H., Hinga M.E., Lobos K.B., Xu Y., Martinez C.P., McCouch S.R. (2003). Mapping quantitative trait loci for yield, yield components and morphological traits in an advanced backcross population between *Oryza rufipogon* and the *Oryza sativa* cultivar Jefferson. Theor. Appl. Genet..

[12] Wang S., Cao M., Ma X., Chen W., Zhao J., Sun C., Tan L., Liu F. (2017). Integrated RNA sequencing and QTL mapping to identify candidate genes from *Oryza rufipogon* associated with salt tolerance at the seedling stage. Front. Plant Sci..

[13] Zheng H., Zhao H., Liu H., Wang J., Zou D. (2015). QTL analysis of Na^+^ and K^+^ concentrations in shoots and roots under NaCl stress based on linkage and association analysis in japonica rice. Euphytica.

[14] Bonilla P., Dvorak J., Mackill D., Deal K., Gregorio G. (2002). RFLP and SSLP mapping of salinity tolerance genes in chromosome 1 of rice (*Oryza sativa* L.) using recombinant inbred lines. Philipp. Agric. Sci..

[15] Gregorio G.B. (1997). Tagging salinity tolerance genes in rice using amplified fragment length polymorphism (AFLP). Ph.D. Thesis.

[16] Ren Z.H., Gao J.P., Li L.G., Cai X.L., Huang W., Chao D.Y., Zhu M.Z., Wang Z.Y., Luan S., Lin H.X. (2005). A rice quantitative trait locus for salt tolerance encodes a sodium transporter. Nat. Genet..

[17] Collins N.C., Tardieu F., Tuberosa R. (2008). Quantitative trait loci and crop performance under abiotic stress: Where do we stand?. Plant Physiol..

[18] Swamy B.P.M., Vikram P., Dixit S., Ahmed H.U., Kumar A. (2011). Meta-analysis of grain yield QTL identified during agricultural drought in grasses showed consensus. BMC Genom..

[19] Goffinet B., Gerber S. (2000). Quantitative trait loci: A meta-analysis. Genetics.

[20] Veyrieras J.B., Goffinet B., Charcosset A. (2007). MetaQTL: A package of new computational methods for the meta-analysis of QTL mapping experiments. BMC Bioinform..

[21] Sosnowski O., Charcosset A., Johann Joets J. (2012). BioMercator V3: An upgrade of genetic map compilation and quantitative trait loci meta-analysis algorithms. Bioinformatics.

[22] Swamy B.P.M., Sarla N. (2011). Meta-analysis of yield QTLs derived from inter-specific crosses of rice reveals consensus regions and candidate genes. Plant Mol. Biol. Rep..

[23] Wu Y., Huang M., Tao X., Guo T., Chen Z., Xiao W. (2016). Quantitative trait loci identification and meta-analysis for rice panicle-related traits. Mol. Genet. Genom..

[24] Ahmadi J., Mohammad F. (2011). Identification and mapping of quantitative trait loci associated with salinity tolerance in rice (*Oryza sativa*) using SSR markers. Iran. J. Biotechnol..

[25] Puram V.R.R., Ontoy J., Subudhi P.K. (2018). Identification of QTLs for salt tolerance traits and prebreeding lines with enhanced salt tolerance in an introgression line population of rice. Plant Mol. Biol. Rep..

[26] Zhou Y., Yang P., Cui F., Zhang F., Luo X., Xie J. (2016). Transcriptome analysis of salt stress responsiveness in the seedlings of Dongxiang wild rice (*Oryza rufipogon* Griff.). PLoS ONE.

[27] Formentin E., Sudiro C., Perin G., Riccadonna S., Barizza E., Baldoni E., Lavezzo E., Stevanato P., Sacchi G.A., Fontana P. (2018). Transcriptome and cell physiological analyses in different rice cultivars provide new insights into adaptive and salinity stress responses. Front. Plant Sci..

[28] Alam R., Sazzadur Rahman M., Seraj Z.I., Thomson M.J., Ismail A.M., Tumimbang-Raiz E., Gregorio G.B. (2011). Investigation of seedling-stage salinity tolerance QTLs using backcross lines derived from *Oryza sativa* L. Pokkali. Plant Breed..

[29] Lin H.X., Zhu M.Z., Yano M., Gao J.P., Liang Z.W., Su W.A., Hu X.H., Ren Z.H., Chao D.Y. (2004). QTLs for Na^+^ and K^+^ uptake of the shoots and roots controlling rice salt tolerance. Theor. Appl. Genet..

[30] Sabouri H., Rezai A.M., Moumeni A., Kavousi A., Katouzi M., Sabouri A. (2009). QTLs mapping of physiological traits related to salt tolerance in young rice seedlings. Biol. Plant..

[31] Price A.H. (2006). Believe it or not, QTLs are accurate!. Trends Plant Sci..

[32] Arcade A., Labourdette A., Falque M., Mangin B., Chardon F., Charcosset A., Joets J. (2004). BioMercator: Integrating genetic maps and QTL towards discovery of candidate genes. Bioinformatics.

[33] Courtois B., Ahmadi N., Khowaja F., Price A.H., Rami J.F., Frouin J., Hamelin C., Ruiz M. (2009). Rice root genetic architecture: Meta-analysis from a drought QTL database. Rice.

[34] Griffiths S., Simmonds J., Leverington M., Wang Y., Fish L., Sayers L., Alibert L., Orford S., Wingen L., Herry L. (2009). Meta-QTL analysis of the genetic control of ear emergence in elite European winter wheat germplasm. Theor. Appl. Genet..

[35] Li W.T., Liu C., Liu Y.X., Pu Z.E., Dai S.F., Wang J.R., Lan X.J., Zheng Y.L., Wei Y.M. (2013). Meta-analysis of QTL associated with tolerance to abiotic stresses in barley. Euphytica.

[36] Shinozuka H., Cogan N.O.I., Spangenberg G.C., Forster J.W. (2012). Quantitative trait locus (QTL) meta-analysis and comparative genomics for candidate gene prediction in perennial ryegrass (*Lolium perenne* L.). BMC Genet..

[37] Zhang H., Uddin M.S., Zou C., Xie C., Xu Y., Li W.X. (2014). Meta-analysis and candidate gene mining of low-phosphorus tolerance in maize. J. Integr. Plant Biol..

[38] Agrama H.A.S., Moussa M.E. (1996). Mapping QTLs in breeding for drought tolerance in maize (*Zea mays* L.). Euphytica.

[39] Wu X.L., Hu Z.L., Rifkin S.A. (2012). Meta-analysis of QTL mapping experiments. Quantitative Trait Loci (QTL): Methods and Protocols.

[40] Khan A., Li R.J., Sun J.T., Ma F., Zhang H.X., Jin J.H., Ali M., Haq S.U., Wang J.E., Gong Z.H. (2018). Genome-wide analysis of dirigent gene family in pepper (*Capsicum annuum* L.) and characterization of CaDIR7 in biotic and abiotic stresses. Sci. Rep..

[41] Ahmadi N., Negrão S., Katsantonis D., Frouin J., Ploux J., Letourmy P., Droc G., Babo P., Trindade H., Bruschi G. (2011). Targeted association analysis identified japonica rice varieties achieving Na^+^/K^+^ homeostasis without the allelic make-up of the salt tolerant *indica* variety Nona Bokra. Theor. Appl. Genet..

[42] Chen X., Wang Y., Lv B., Li J., Luo L., Lu S., Zhang X., Ma H., Ming F. (2014). The NAC family transcription factor OsNAP confers abiotic stress response through the ABA pathway. Plant Cell Physiol..

[43] Gupta B., Huang B. (2014). Mechanism of salinity tolerance in plants: Physiological, biochemical, and molecular characterization. Intl. J. Genom..

[44] Li W.T., He M., Wang J., Wang Y.P. (2013). Zinc finger protein (ZFP) in plants-a review. Plant Omics.

[45] Hossain M.R., Bassel G.W., Pritchard J., Sharma G.P., Ford-Lloyd B.V. (2016). Trait specific expression profiling of salt stress responsive genes in diverse rice genotypes as determined by modified significance analysis of microarrays. Front. J. Plant Sci..

[46] Diédhiou C.J., Popova O.V., Dietz K.J., Golldack D. (2008). The SNF1-type serine-threonine protein kinase SAPK4regulates stress-responsive gene expression in rice. BMC Plant Biol..

[47] Ouyang S.Q., Liu Y.F., Liu P., Lei G., He S.J., Ma B., Zhang W.K., Zhang J.S., Chen S.Y. (2010). Receptor-like kinase *OsSIK1* improves drought and salt stress tolerance in rice (*Oryza sativa*) plants. Plant J..

[48] Tanaka H., Osakabe Y., Katsura S., Mizuno S., Maruyama K., Kusakabe K., Mizoi J., Shinozaki K., Yamaguchi-Shinozaki K. (2012). Abiotic stress-inducible receptor-like kinases negatively control ABA signaling in Arabidopsis. Plant J..

[49] Rosas-Santiago P., Lagunas-Gómez D., Barkla B.J., Vera-Estrella R., Lalonde S., Jones A., Frommer W.B., Zimmermannova O., Sychrova H., Pantoja O. (2015). Identification of rice cornichon as a possible cargo receptor for the Golgi-localized sodium transporter OsHKT1;3. J. Exp. Bot..

[50] McCouch S.R., Teytelman L., Xu Y., Lobos K.B., Clare K., Walton M., Fu B., Maghirang R., Li Z., Xing Y. (2002). Development and mapping of 2240 new SSR markers for rice (*Oryza sativa* L.). DNA Res..

[51] Darvasi A., Soller M. (1997). A simple method to calculate resolving power and confidence interval of QTL map location. Behav. Genet..

[52] IRGSP (2005). The map-based sequence of the rice genome. Nature.

[53] Tian T., Liu Y., Yan H., You Q., Yi X., Du Z., Xu W., Su Z. (2017). agriGO v2.0: A GO analysis toolkit for the agricultural community, 2017 update. Nucleic Acids Res..

[54] Gregorio G., Senadhira D., Mendoza R. (1997). Screening Rice for Salinity Tolerance.

[55] Jones J.B., Case V.W., Westerman R.L. (1990). Sampling, handling, and analyzing plant tissue samples. Soil Testing and Plant Analysis.

[56] Chen D.H., Ronald P.C. (1999). A rapid DNA minipreparation method suitable for AFLP and other applications. Plant Mol. Biol. Rep..

