# Supplementary material for: Meta-Analysis of Quantitative Trait Loci Associated with Seedling-Stage Salt Tolerance in Rice (Oryza sativa L.)
Source: Plants (Basel). 2019 Jan 29;8(2):33. doi: 10.3390/plants8020033 (PMC6409918; doi:10.3390/plants8020033)
Supplement: Supplementary file 1 [file plants-08-00033-s001.zip › Table S5 Summary of the integrated consensus map.docx]

**Table S5.** Summary of the integrated consensus map.

| Chromosome | Number of markers | Number of QTLs | Length (cM) | Mean distance of two markers (cM) |
| --- | --- | --- | --- | --- |
| 1 | 1,709 | 23 | 365 | 0.21 |
| 2 | 1,328 | 18 | 331.26 | 0.25 |
| 3 | 1,382 | 10 | 347.83 | 0.25 |
| 4 | 1,103 | 11 | 261.6 | 0.24 |
| 5 | 1,012 | 12 | 236.7 | 0.23 |
| 6 | 1,103 | 6 | 262.69 | 0.24 |
| 7 | 993 | 4 | 220.37 | 0.22 |
| 8 | 693 | 5 | 206.55 | 0.30 |
| 9 | 762 | 5 | 153.52 | 0.20 |
| 10 | 639 | 6 | 156.78 | 0.25 |
| 11 | 799 | 7 | 209.26 | 0.26 |
| 12 | 804 | 8 | 184.37 | 0.23 |
| Total | 12,327 | 115 | 2,935.93 | 0.24 |

cM, Centi Morgan
