# Supplementary material for: Meta-Analysis of Quantitative Trait Loci Associated with Seedling-Stage Salt Tolerance in Rice (Oryza sativa L.)
Source: Plants (Basel). 2019 Jan 29;8(2):33. doi: 10.3390/plants8020033 (PMC6409918; doi:10.3390/plants8020033)
Supplement: Supplementary file 1 [file plants-08-00033-s001.zip › Table S8 List of significant GO terms.docx]

**Table S8.** List of significant GO terms obtained in the five meta-QTL regions for seedling stage salt tolerance.

| Meta-QTLs | GO term | Ontology^*^ | GO term name | Number in input list^ǂ^ | Number of genes in reference database^ψ^ | p-value^φ^ | FDR^**^ |
| --- | --- | --- | --- | --- | --- | --- | --- |
| *MQTLSIS1.4* | GO:0008168 | F | methyltransferase activity | 6 | 261 | 0.00013 | 0.0091 |
|  | GO:0016741 | F | transferase activity, transferring one-carbon groups | 6 | 263 | 0.00013 | 0.0091 |
| *MQTLSNK2.1* | GO:0009141 | P | nucleoside triphosphate metabolic process | 6 | 135 | 0.00064 | 0.027 |
|  | GO:0009142 | P | nucleoside triphosphate biosynthetic process | 6 | 132 | 0.00057 | 0.027 |
|  | GO:0009144 | P | purine nucleoside triphosphate metabolic process | 6 | 134 | 0.00062 | 0.027 |
|  | GO:0009145 | P | purine nucleoside triphosphate biosynthetic process | 6 | 132 | 0.00057 | 0.027 |
|  | GO:0009205 | P | purine ribonucleoside triphosphate metabolic process | 6 | 134 | 0.00062 | 0.027 |
|  | GO:0009199 | P | ribonucleoside triphosphate metabolic process | 6 | 134 | 0.00062 | 0.027 |
|  | GO:0009206 | P | purine ribonucleoside triphosphate biosynthetic process | 6 | 132 | 0.00057 | 0.027 |
|  | GO:0009201 | P | ribonucleoside triphosphate biosynthetic process | 6 | 132 | 0.00057 | 0.027 |
|  | GO:0046034 | P | ATP metabolic process | 6 | 129 | 0.00051 | 0.027 |
|  | GO:0006754 | P | ATP biosynthetic process | 6 | 127 | 0.00047 | 0.027 |
|  | GO:0009259 | P | ribonucleotide metabolic process | 6 | 151 | 0.0011 | 0.032 |
|  | GO:0009150 | P | purine ribonucleotide metabolic process | 6 | 147 | 0.00098 | 0.032 |
|  | GO:0009152 | P | purine ribonucleotide biosynthetic process | 6 | 145 | 0.00091 | 0.032 |
|  | GO:0009260 | P | ribonucleotide biosynthetic process | 6 | 149 | 0.001 | 0.032 |
|  | GO:0006164 | P | purine nucleotide biosynthetic process | 6 | 150 | 0.0011 | 0.032 |
|  | GO:0006163 | P | purine nucleotide metabolic process | 6 | 154 | 0.0012 | 0.033 |
|  | GO:0005515 | F | protein binding | 38 | 2460 | 1.20E-05 | 0.0035 |
|  | GO:0042625 | F | ATPase activity, coupled to transmembrane movement of ions | 6 | 94 | 0.0001 | 0.015 |
|  | GO:0015405 | F | P-P-bond-hydrolysis-driven transmembrane transporter activity | 7 | 170 | 0.00035 | 0.026 |
|  | GO:0015399 | F | primary active transmembrane transporter activity | 7 | 170 | 0.00035 | 0.026 |
|  | GO:0042626 | F | ATPase activity, coupled to transmembrane movement of substances | 6 | 142 | 0.00082 | 0.041 |
|  | GO:0043492 | F | ATPase activity, coupled to movement of substances | 6 | 142 | 0.00082 | 0.041 |
|  | GO:0042623 | F | ATPase activity, coupled | 8 | 275 | 0.0012 | 0.041 |
|  | GO:0016820 | F | hydrolase activity, acting on acid anhydrides, catalyzing transmembrane movement of substances | 6 | 147 | 0.00098 | 0.041 |
|  | GO:0017111 | F | nucleoside-triphosphatase activity | 14 | 726 | 0.0012 | 0.041 |
|  | GO:0016462 | F | pyrophosphatase activity | 14 | 743 | 0.0015 | 0.045 |
|  | GO:0016818 | F | hydrolase activity, acting on acid anhydrides, in phosphorus-containing anhydrides | 14 | 759 | 0.0019 | 0.049 |
|  | GO:0016817 | F | hydrolase activity, acting on acid anhydrides | 14 | 764 | 0.002 | 0.049 |
| *MQTLSNK2.2* | GO:0071669 | P | plant-type cell wall organization or biogenesis | 6 | 34 | 3.60E-06 | 0.00064 |
|  | GO:0009664 | P | plant-type cell wall organization | 6 | 34 | 3.60E-06 | 0.00064 |
|  | GO:0071555 | P | cell wall organization | 6 | 83 | 0.00035 | 0.042 |
|  | GO:0045735 | F | nutrient reservoir activity | 8 | 77 | 3.10E-06 | 0.0011 |
|  | GO:0005515 | F | protein binding | 49 | 2460 | 1.40E-05 | 0.0023 |
| *MQTLSNK2.3* | GO:0004607 | F | phosphatidylcholine-sterol O-acyltransferase activity | 8 | 24 | 8.70E-07 | 0.00047 |
|  | GO:0008374 | F | O-acyltransferase activity | 8 | 33 | 6.50E-06 | 0.0018 |
|  | GO:0004722 | F | protein serine/threonine phosphatase activity | 8 | 54 | 0.00014 | 0.026 |
| *MQTLSNK2.4* | GO:0043229 | C | intracellular organelle | 7 | 2859 | 0.0014 | 0.018 |
|  | GO:0043226 | C | organelle | 7 | 2859 | 0.0014 | 0.018 |
|  | GO:0005622 | C | intracellular | 8 | 4116 | 0.0026 | 0.022 |
|  | GO:0044424 | C | intracellular part | 7 | 3529 | 0.0049 | 0.025 |
|  | GO:0005623 | C | cell | 9 | 5945 | 0.0069 | 0.025 |
|  | GO:0005634 | C | nucleus | 5 | 1884 | 0.0062 | 0.025 |
|  | GO:0044464 | C | cell part | 9 | 5945 | 0.0069 | 0.025 |
|  | GO:0043227 | C | membrane-bounded organelle | 5 | 2319 | 0.015 | 0.041 |
|  | GO:0043231 | C | intracellular membrane-bounded organelle | 5 | 2296 | 0.014 | 0.041 |

^*^F, molecular function; P, biological process; C, cellular component

^ǂ^Number of annotated genes in the query list under each GO term

^Ψ^Number of genes available in the reference database under each GO term

^φ^P-value indicate the significance level

^**^FDR, False discovery rate under dependency of multi-test adjustment method
