# Supplementary figures and images for: Meta-Analysis of Quantitative Trait Loci Associated with Seedling-Stage Salt Tolerance in Rice (Oryza sativa L.)
Source: Plants (Basel). 2019 Jan 29;8(2):33. doi: 10.3390/plants8020033 (PMC6409918; doi:10.3390/plants8020033)

[illegible]

consensus



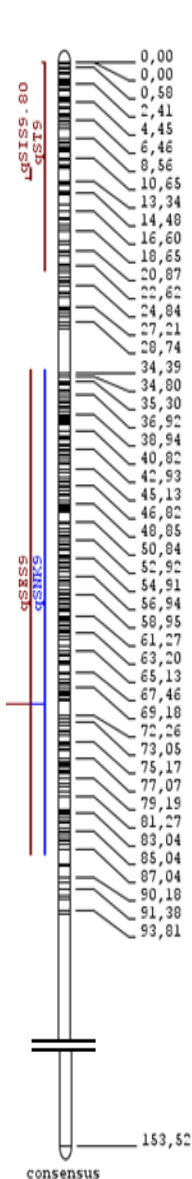

9

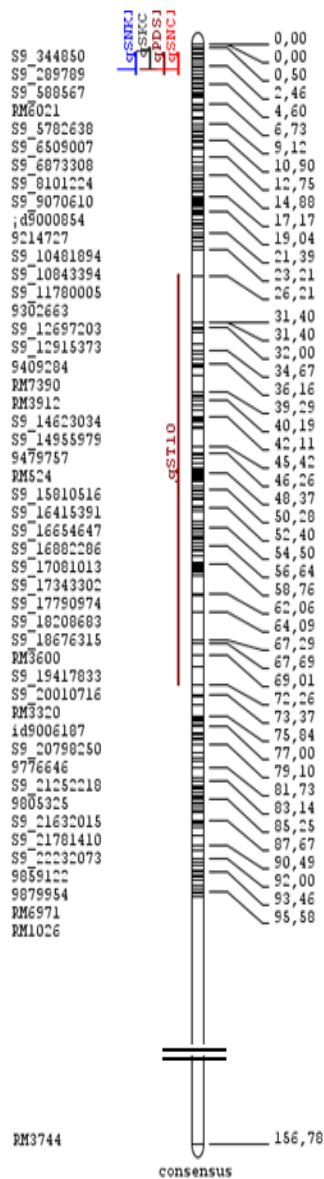

10

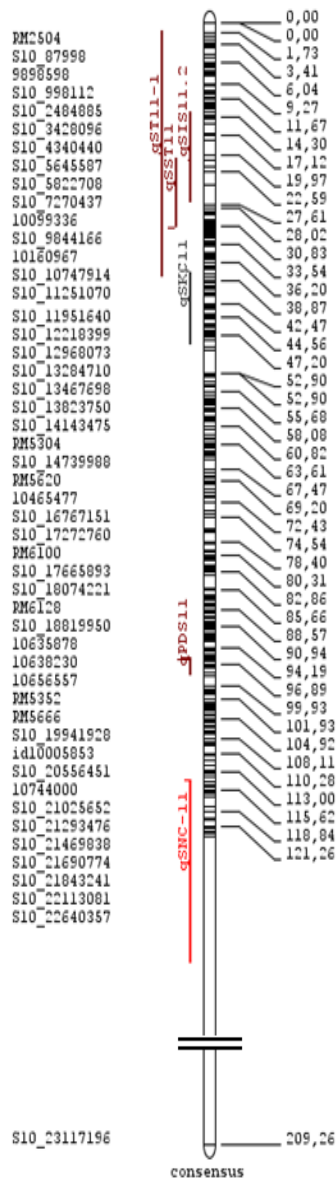

11

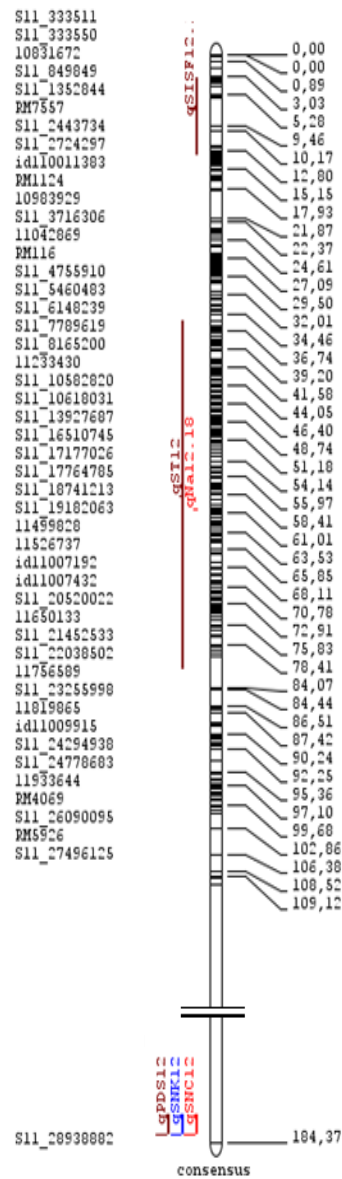

12

Supplement: Supplementary file 1 [file plants-08-00033-s001.zip › Figure S1 The integrated consensus map.pdf]

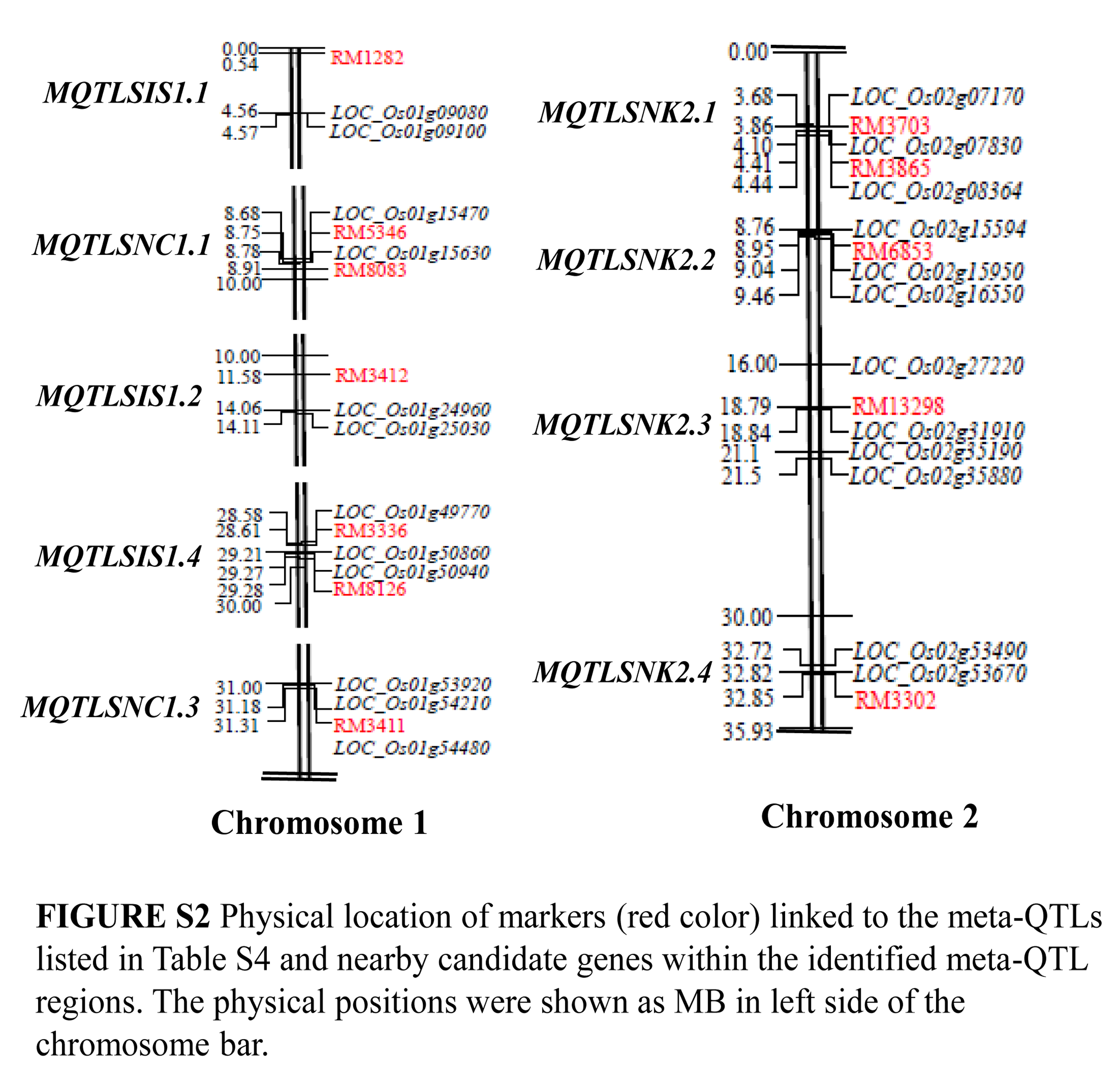

Supplement: Supplementary file 1 [file plants-08-00033-s001.zip › Figure S2 physical location of markers.tif]

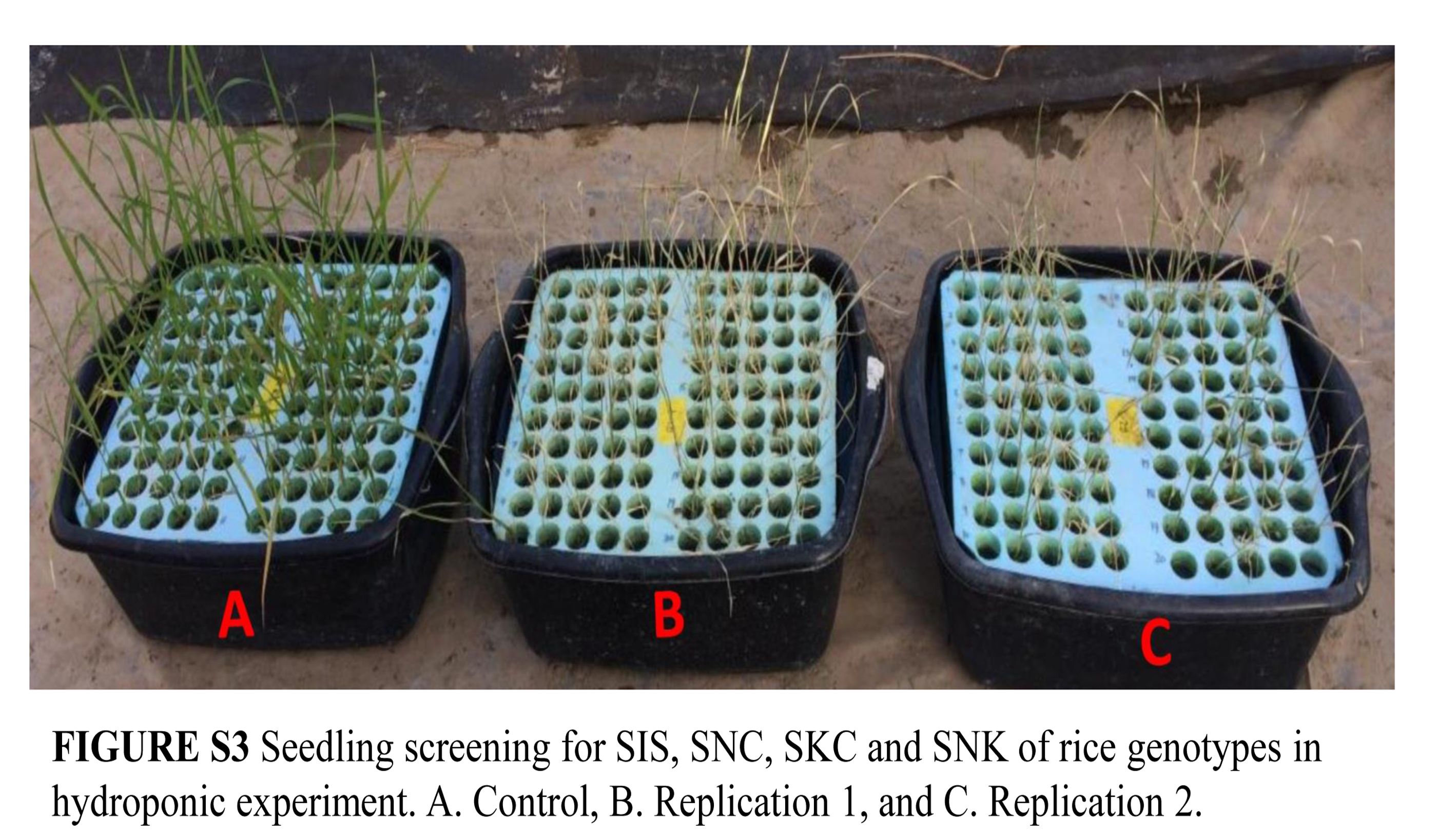

Supplement: Supplementary file 1 [file plants-08-00033-s001.zip › Figure S3 hydroponic expt.tif]
